# Comparison of Conformation and Movement Characteristics in Dressage and Jumping Sport Warmblood Mares Based on Point Evaluation and Linear Scoring System

**DOI:** 10.3390/ani13193101

**Published:** 2023-10-04

**Authors:** Alicja Borowska, Dorota Lewczuk

**Affiliations:** 1 Faculty of Veterinary Medicine and Animal Science, Poznan University of Life Sciences, ul. Wolynska 33, 60-637 Poznan, Poland; aborek@up.poznan.pl; 2 Institute of Genetics and Animal Biotechnology PAS, ul. Postepu 36A, 05-552 Magdalenka, Poland

**Keywords:** sport horse, discipline type, evaluation system, linear profiling, conformation

## Abstract

**Simple Summary:**

Conformation assessment is the basis for the selection of horses for a specific sport. Traditional 100-point judging systems, linear scoring and basic measurements are simultaneously used in Poland for evaluation of the same warmblood sport horses. This study aimed to compare traditional 100-point judging systems and linear profiling and to find characteristic traits of specific sport types by analyzing the differences between warmblood mares that are qualified as dressage vs. jumping. The research showed that warmblood mares classified as different types varied in a few linear conformation traits, most significantly in the position of the shoulder, line of the loins or shape of the croup. Analyses of linear movement traits showed that dressage warmblood mares moved better in walking and more balanced canter than jumping ones. These differences were not evident in the overall 100-point system. Both point-scaling systems were correlated only between the same named traits—type, movement and gaits. Finding detailed, measured, objective characteristics for various sport types is important for breeding and judging.

**Abstract:**

The aim of this study was to analyze the influence of factors on the results of 100-point judging systems, linear scoring and basic measurements, as well as differences between systems for dressage and jumping warmblood mares. The research covered official data on 1547 warmblood mares. Analysis of variance and phenotypic correlations (Pearson and partial) were used. The analysis showed that sport type significantly influenced 1/3 of biometric measurements, 2/9 traits on the 100-point system and 7/37 of linear scored traits. The influence of horse type evaluation is more significant in linear scoring than in the 100-point evaluation, which provides an argument for using the first system in breeding. In the linear evaluation for warmblood mares grouped as jumping or dressage, the most significant differences (*p* < 0.001) were noted between the traits of shoulder position, line of the loins and shape of the croup. In the point-based evaluation, differences (*p* < 0.05) were found in forelimbs and walking, as well as chest circumference in basic measurements. None of the traits in the two evaluation systems is identical to any other (r > 0.8). The comparison of systems showed meaningful correlations only between general traits. The differences between sport types of warmblood mares were smaller than expected. More objective traits should be discovered to increase accuracy in discriminating between horse types.

## 1. Introduction

Selection of horses is based primarily on the correctness of their conformation and movement. Conformation traits are often used as indicator traits for performance, since conformation traits are more heritable and are connected to the genetic background of performance [1]. Linear profiling is currently one of the best-known evaluation systems being introduced for many different breeds [1,2] of different statuses, such as, e.g., sport horses [3,4], coldblooded horses [5] or endangered breeds [6,7]. The leading breeding associations on the equine market, such as the Dutch (KWPN) or Belgian (BWP) warmblood horse-breeding societies, have implemented the so-called linear evaluation of traits for breeding horses, taken from cattle breeding [2], in view of its successful application in dairy cattle [8], sheep [9] and pigs [10]. This linear profiling makes it possible for genetic progress to be a successive breeding tool to assess conformation with a satisfactory heritability level [3]. Many countries maintain linear evaluation for sport horses, but many other breeds, such as Irish draught horses, are evaluated using the comparative approach. The first linear evaluation was implemented in the 1980s; traits were chosen for their economic relevance and potential for improving performance [2]. The scaling of traits can be different as well, with −40/+40, 0–40, −3/+3, but “a-i” marks are usually applied as the basic levels. Detecting defects is of great importance in linear scoring; however, in most countries, they are marked as binary traits. Over the years, the methods of evaluation have been assessed, and finally, in many countries, different scales and systems have been adopted. Nowadays, changes in scaling and traits are being introduced, with some being added and some removed. The breeding standards for dressage and showjumping horses have been developed as separate evaluation sheets [11]. Unfortunately, in the case of numerous traits, differences in conformation seem to not be specified enough for the different types of horse use (e.g., body shape in a rectangle; long lines and harmonious proportions; long, raised and rounded neck). It also seems that there is not enough description of gait characteristics. Thus, a lack of differentiation between types of horses can be a problem for judges’ evaluations, especially when horse specialization is becoming more popular [12,13,14]. Horse-breeding societies use semi-objective measures to evaluate conformation and movement [15,16,17]. Objective measures are available [18,19,20,21,22], but they are often difficult to implement within equestrian events.

The traditional points system has been criticized because of the lack of agreement with the information on functional performance [23]. Traditional judging systems express scores in relation to the idea of the trait, whereas linear scoring expresses traits in relation to the population mean based on trait extremes. According to the literature, this approach makes such a system more descriptive, comparable and objective [24]. Linear scoring maximizes information output for breeders, lowering the risk of biases due to personal preferences. However, specificity of definitions and training of assessors is of great importance and should be provided as continuous education. According to Duensing et al. [1], a total of seven to ten scores is optimum for every trait evaluation because of the limited abilities of the human eye. This system seems also natural, as judges usually take personal notes; thus, the scaling is in accordance with their preferences. In Poland, the 100-point evaluation scoring system is the basis for an entry in stud books of warmblood horses. This system assesses the characteristics of horses in points according to the value seen by the evaluator. Advantages of this system include simplicity (a small number of traits), clarity of the assessment (the higher the score, the better) and the general knowledge of the system by breeders and equestrians [25]. The disadvantage of this system is that it is not possible to obtain a sufficient characterization of the horse’s conformation due to the low level of detail, failure to use the full scoring scale and thus the inability to provide sufficient information about the horse being evaluated. This has persuaded breeders to introduce a new linear system. This system changes the qualitative assessment into a descriptive assessment of conformation and movement by the assessor according to a strictly defined scheme on the scale “a–i”, prepared separately for different types of use of horses. Advantages of this scoring system are connected with detailed information and greater objectivity. In turn, disadvantages of this assessment include its complexity (many traits) and a lack of unambiguous information concerning the quality of conformation (only its description is used). For several years in Poland, young horses have been assessed using both scoring systems, which enables a comparison of both evaluation systems for the same horses of different types. The traditional scoring system could detect differences between types of horses [26] but did not differ in the scale of marks allocated. The expected differences between horses qualified as dressage (DH) vs. jumping (JH) would be of special value. The correlations between traits of both systems were also analyzed. Finding differences in the sport type of horses is of special importance for future judging in the case of less experienced experts or horse beginners [24]. According to our expectations, horses should differ in at least half of the evaluated traits. The aim of the study was to analyze the influence of genetic and environmental factors on the results of 100-point evaluation, linear scoring and basic measurements for warmblood mares, as well as to assess the relationship between the registered traits within each scoring system and between them.

## 2. Material and Methods

The research covered data on 1547 warmblood mares subjected to a 100-point evaluation and linear profiling during performance tests or horse inspection. The results were collected from the Polish Horse Breeders Association (PHBA) horse database. All available traits of the conformation and movement assessment as well as biometric measurements of mares were analyzed. Basic measurements consisted of height at the withers and circumferences of the cannon bone and chest (cm) (Supplementary Table S1). The traditional 100-point system consists of the following traits [26]: type (0–15 pts), head and neck (0–5 pts), body (0–10 pts), front legs (0–10 pts), hind legs (0–10 pts), hooves (0–10 pts), walk (0–10 pts), trot (0–10 pts) and general impression (0–15 pts) (Supplementary Table S2). 

The linear scoring system consists of the following groups of traits [11]:

1. Overall evaluation (also on a 0–10 point scale) of movement, conformation, type, walk, trot, canter, free-jumping, reflex, technique, abilities (Supplementary Table S3);
2. Conformation traits—linear description (“a–i”—9 alphabetical marks) of body shape, body direction, head–neck connection, length of neck, position of neck, muscling of neck, height at the withers, length of withers, position of shoulder, line of back, line of loins, shape of croup, length of croup, stance of forelegs, stance of hind legs, stance of front pastern, stance of hind pastern, shape of hooves, heels, quality of legs, substance of legs (Supplementary Table S4);
3. Movement traits—linear description (“a–i”—9 alphabetical marks) of movement traits: length of stride, correctness of walk, length of stride, elasticity, impulsion, balance in trot and canter (Supplementary Table S5);
4. Jumping traits—linear description (“a–i”—9 alphabetical marks) of jumping traits: take-off direction, take-off quickness, foreleg technique, back technique, haunch technique, scope, elasticity, care, engagement (Supplementary Table S6).

In both systems, horses were presented in hand and additionally in free jumping in linear scoring. Basic conformation was evaluated in a standing position on a hard surface; walk and trot were then shown in hand on a hard surface. The free jumping was presented in the riding hall. The free jumping consisted of a line of obstacles (pole on the ground; then, at a distance of 2.5–3 m, a vertical obstacle 60 cm high; then, at a distance of 6.8–7.2 m, a spread obstacle up to 100 cm height and 80 cm width). Horses moved freely in all gaits in both directions, then were led to the jumping line. All evaluations were conducted by at least three trained judges. Detailed information on the evaluation system can be found at breeders’ associations websites (https://www.wbfsh.com/linear-scoring (accessed on 30 September 2023); https://equnephenoypes.org/Texte/home_ENG.html (accessed on 30 September 2023); https://www.pzhk.pl/hodowla/programy-hodowlane (accessed on 30 September 2023).

The analysis includes all available Polish Horse Breeders Association data from warmblood mares, with conformation and movement assessment according to both systems from 2014 to 2019. Despite a large amount of material collected (on more than 1500 mares), only about 600 mares were scored for canter, and about 130 completed jump information. The number of warmblood mares assessed as jumping mares is very small—less than 10% of the estimated horse population.

Warmblood mares were divided into different sport types—dressage (986 mares) and jumping (561 mares)—by their owners, who participated in the linear evaluation. The breed structure of the analyzed population was as follows: 401 Malopolski horses (m), 375 Wielkopolski horses (wlkp), 579 Polish sport horses (sp) and 188 other foreign breeds. The breeds are closely genetically connected with each other. The mares came from 754 sires and were judged in 16 districts. The following statistical analyses were used to compare the investigated scoring systems:

- Analysis of variance of the traits registered using both systems, with a comparison of the impact of selected factors (sport type classification, assessment district, breed, sire effect) on the results;
- Analysis of phenotypic Pearson correlations between traits in the 100-point evaluation and linear scoring system;
- Analysis of phenotypic partial correlations between traits in the 100-point evaluation and linear scoring system using the statistical model described above for analysis of variance.

The analyses of the values of traits in linear profiling were transformed according to Dutch authors [27,28], as the linear scoring system in Poland is based on the Dutch system. The descriptive letter marks (“a–i”) were converted into a scale of 1–40 points, with 20 points corresponding to the average value. The UNIVARIATE procedure was used for analysis of the data distribution. The basic statistics information (mean, standard deviation, minimum and maximum) of the data was achieved using the MEANS procedure (SAS software, ver. 9.4, Cary, NC, USA: SAS Institute Inc., 2013).

### 2.1. Analysis of Variance of Horse Basic Measurments, Linear Profiling and 100-Point Evaluation

The mixed linear model included the sport type (1—dressage and 2—jumping mares), district (1–16 according to breeders’ organizations), breed register (1–4 as described above) and sire effect used in the statistical analysis (754). The data are distributed through different factors. All type of mares were present in all breeds; 60/64 breed-district classes are observed as well as 8/32 type-district classes. The influence of the sire as a random factor was expressed by the repeatability of the trait (which is the maximum possible heritability of the trait when estimated by the paternal model) calculated from the random effects (the ratio of the sire effects to all random effects—sire and residual). Other factors, as fixed effects, were expressed in terms of statistical significance (*p*-values). All identified factors that can influence the results were used.

The MIXED procedure in SAS software (ver. 9.4, 2013) was used for the analysis of variance according to the statistical model (as described above):

y_ijklm_ = s_i_ + T_j_ + D_k_ + B_l_ + e_ijklm_ (1)

where y_ijklm_—mean of the trait; s_i_—random sire effect (1–754); T_j_—fixed effect of the horse type (1,2); D_k_—fixed effect of the breeding district (1–16); B_l_—fixed effect of the breed register (1,2,3,4); e_ijklm_—random residual effect.

### 2.2. Pearson Correlations between Traits in the 100-Point Evaluation and Linear Scoring System 

The Pearson values were calculated using the CORR procedure of the SAS software (version 9.4, 2013) between 9 traits evaluated in the 100-point evaluation (type, head and neck, body, front legs, hind legs, hooves, walk, trot, general impression) and the linear scoring traits (10 general, 21 conformation, 10 movement, 9 jumping traits). Results were presented as significant for *p* < 0.05.

### 2.3. Partial Correlations between Traits in the 100-Point Evaluation and Linear Scoring System 

The GLM procedure with the Manova option was used for calculations of the partial correlations to study connections between traits corrected for all significant effects. The same statistical model was used (described above). Partial correlations were calculated between 9 traits assessed in the 100-point evaluation (type, head and neck, body, front legs, hind legs, hooves, walk, trot, general impression) and linear scoring traits (10 general, 21 conformation, 10 movement, 9 jumping traits). Results were presented as significant for *p* < 0.05.

## 3. Results

### 3.1. Trait Variability

The means for biometric measurements were 164.4 ± 4.4 cm for height at the withers, 193.9 ± 7.89 cm for chest circumference and 20.5± 0.92 cm for cannon circumference. The coefficient of variation was very low for these traits (4%). Statistical characteristics of traits in both evaluation systems are presented in Table 1, Table 2 and Table 3. The conformation 100-point evaluation showed a greater variability, from 4 to 10% (mean 7%). The highest value was obtained for the “head” trait, and the lowest was recorded for the “type” and “overall” traits. 

Linear scoring of traits was characterized by greater variability of between 12 and 42% (mean 31%) depending on the group of the traits. As a part of the linear scoring sheet, overall points fell into the 10–15% range, with the highest for jumping traits. The same tendency was observed for descriptive linear scored traits, with jumping being higher (42%) than for movement (35%) and body conformation (33%). The narrowest scale of evaluation was observed for the two traits of the “length of the neck” and the “hind pastern” (seven grades without extremes).

A lack of extreme values was observed for 23 of 39 traits (Table 3). Eleven traits were noted without the highest extreme value (i.e., 40), and sixteen traits were noted without the lowest extreme value (i.e., 1). 

### 3.2. Biometric Measurements

The analysis showed that the dressage and show-jumping warmblood mares differed only in chest circumference (*p* = 0.0013). On the other hand, a high statistically significant influence (*p* < 0.0003) was demonstrated for the district (*p* < 0.0003) and breed (*p* < 0.0001; Table S7) in the case of all traits. 

The repeatability coefficients took average values only for height at the withers (0.30), whereas for both other measurements, the values were low (0.13–0.19). The analysis of the differences between the sport types showed that the jumping mares had a smaller circumference by about 2 cm (Table 4).

### 3.3. Traditional 100-Point Scoring Assessment

The analysis of the studied factors showed that the sport type of the mares did not have an influence on recorded traits in the 100-point evaluation system, except for forelegs (*p* = 0.0274) and walk (0.0368). The repeatability coefficients of the scored traits are low (0.02–0.11). The differences between the breeds are statistically important (*p* < 0.03) for type, hind legs, walk, trot and general impression (Table S8). On the other hand, the district effect (*p* < 0.0001) is significant for all the traits and is greater than the effect of breed (*p*-value between non-significant and <0.0001). Repeatability for all traits was low (0.02–0.11).

The assessment results for dressage-type warmblood mares were mostly higher than those for show-jumping mares (except for trot), but they were significant only for the forelegs and walk. The standard error (indicating diversity of the population) was higher for the jumper group for walk (Table 5).

### 3.4. Linear Scoring System

#### 3.4.1. General Traits 

The analysis showed no significant influence of the sport type classification on the general traits in linear profiling, whereas the district (*p* < 0.0001) is a significant factor except for jumping marks. Similar results were observed for the breed effect (*p* < 0.0012) except for walk (Table S9). The repeatability of general traits was low (0.12–0.30) for the movement traits and medium (0.36–0.51) for the scores of jumping traits, despite the small size of the examined jumping population. The lowest repeatability coefficient was obtained for the general conformation mark (0.12). No analysis of sport type effects for jumping general traits was conducted. This was due to the small number of mares qualified as dressage mares that had their jumping traits assessed. The analysis of sport type effects for the movement traits is presented in the Table 6. There are no differences between movement traits for jumping and dressage types of mares. 

#### 3.4.2. Conformation Traits 

The analysis showed that show-jumping and dressage mares differed in terms of the neck position (*p* = 0.0373), shoulder position (*p* = 0.0036), line of the loins (*p* = 0.0142), shape of the croup (*p* = 0.0054), stance of the front pastern (*p* = 0.0266) and quality of the legs (*p* = 0.0440). On the other hand, the district had a statistically significant influence (*p* ≤ 0.0001) on all traits. In turn, the breed effect was found for body shape (*p* = 0.0051), body direction (*p* = 0.0002) and all the traits of the neck structure (*p*-value between 0.0002 and 0.046) (Table S10). In addition, warmblood mares of various breeds differed in the scores for stance of the front pastern (*p* = 0.0353), quality of the legs (*p* ≤ 0.0001) and substance of the legs (*p* = 0.0007). Repeatability of the conformation traits according to the linear evaluation showed very low values, none of which exceeded 0.12, noted for the heels and substance of the legs.

Warmblood mares of various sport types differed in the following conformation traits in the linear assessment: The position of the neck was higher than the average for dressage warmblood mares, as was the position of the shoulder and the shape of the croup. The loin line in these mares was above the average. On the other hand, the values of dressage warmblood mares for the stance of the pastern and the quality of the legs were below the average and lower than in the jumping mares (Table 7).

#### 3.4.3. Movement Traits 

The analysis of movement traits showed the influence of the sport type only on the assessment of the balance of canter (*p* = 0.0103). The district has a similar influence on the movement traits than on the other linear scores—it is a factor that significantly influences most of the scores (*p* < 0.0023), except for the correctness of walk.

The breed was a significant factor in elasticity (*p* = 0.01 for trot, *p* = 0.03 for canter) and balance (*p* = 0.02 for trot, *p* = 0.04 for canter). All details are in Table S11. The repeatability of the movement traits in the linear assessment was higher than that of the conformation traits, but it was still low. The highest values were recorded for canter ratings (0.13–0.19). The detailed values for the only trait that differs between sport types—the balance of canter—is presented below (Table 8). It is on the same side of the mean for both types of mares (above 20).

#### 3.4.4. Jumping Traits 

Only 130 individuals were analyzed for jumping traits, because only this number of warmblood mares had complete jumping marks. In the analysis of these traits, the breed was a non-significant factor (Table S12). The district was statistically important for the evaluation of the back technique (*p* = 0.02) and care (*p* = 0.01).

Repeatability of the jumping traits in the linear assessment was higher (0.1–0.32) than for the other traits in the linear evaluation, reaching a medium–high level for elasticity (0.32) and back technique (0.3). The foreleg technique showed zero repeatability.

### 3.5. Comparison of the Results for 100-Point Score Assessment and Linear Evaluation 

The correlations between the general marks in the linear evaluation and the 100-point score assessment are positive and show a similar direction of the assessment. However, the values of these correlations, especially for the traits with the same names, seem to be somewhat low. The type in the 100-point assessment is related to the type in the linear assessment in more than 0.5, walk in the 100-point scale is related to the linear walk assessment in almost 0.7, and the same is true for trot assessment between the systems. The other 100-point scale traits that are related to the new linear assessments are the type and general impression in the 100-point assessment (Table 9), with most correlations above 0.3. 

The correlations between the 100-point assessments and detailed linear traits of conformation are mostly low and negative (Table 10). The highest correlation was observed between the hoof and hoof shape scores (−0.45). Medium correlations were recorded for the head and neck connection and the head and neck scores; for the position of the shoulder and type (−0.24); for the body and neck muscles (−0.22), the back line (−0.25), the loin line (−0.27), the croup length (−0.26) and the general impression and neck muscles (−0.24). 

Higher correlations were obtained for movement traits and 100-point assessments (Table 11). Type is correlated with 8 of the 10 movement traits, ranging from −0.21 to −0.28. Specific traits of movement correlated with walk score (−0.66) and trot score (−0.47 to −0.60).

The jump traits were associated with the movement traits (Table 12). Only elasticity of the jump was related to walk (−0.25), and trot was related to the direction of take off (−0.20), scope (−0.30) and the haunch technique (−0.20). Correlations of about −0.3–−0.2 were also recorded between the type and jumping traits and the body and jumping traits, and several jumping traits were related to the head and neck (take off: quickness, forelegs and back technique). The overall impression was related to all the jumping traits except for the foreleg technique.

Partial correlations (phenotypic correlations corrected for the significant factors from the analysis of variance) showed a lower number of significant connections between traits. General linear scoring and 100-point assessment were correlated mostly between the trait type evaluated in points and general traits in linear scoring (except jumping evaluation) at about 0.51–0.64. Some middle–high connections were found between conformation/type evaluated in points and hind legs and hooves evaluated in linear scoring (0.53–0.71). Single correlations were noted between trot in points and linear evaluation of conformation (0.53), general impression in points and conformation (0.54) and general impression in points and linear evaluated trot (0.48). For general traits, for a possible 90 connections, 13 were statistically significant, being below 0.8. The connections between movement and jumping traits were calculated only for walk in points and walk correctness evaluated linearly (−0.67), hind legs in points and trot balance (−0.57) as well as trot in points and foreleg technique evaluated linearly (0.50). 

## 4. Discussion

### 4.1. Trait Variability

The observed variability of traits supports findings reported earlier [2,4,23]—jumping traits are more variable traits, as well as all linear scored traits, which received higher values. However, the number of traits lacking extremes (23 cases for 49) can lead to the deduction that the scale used by KWPN is not quite adequate for the Polish sport horse population, or that the assessment and trait definition are not adequately comparable. Standard deviations and the range of traits were lower than expected for linear traits. This is probably why it was suggested that the traits with the optimum in the middle can be divided into two traits describing the position in relation to the extreme [23]. Such a description and division of traits with the optimum in the middle into two traits according to the extreme was suggested by Viklund and Eriksson [4]. A linear scale is mostly considered as a linear continuous scale, so the traits can be analyzed by mixed-linear models, as nowadays, these are a standard tool to estimate the unbiased estimated breeding value [3]. 

### 4.2. Sport Type Differences

The sport type effect was not remarkable for any of the scaling used. Basic measurements were different only for one of three basic parameters—warmblood mares classified as the dressage type were 1% larger (percentage counted from the smaller value) in the chest circumference. A higher body mass, connected with the circumference of the chest, was also found in dressage horses compared to jumping ones [29]. The 100-point scaling showed that dressage mares were evaluated higher in two of ten traits (1.4% in front limb conformation and 1.2% in walk). Differences between front limb conformation between jumping and dressage horses were found for adult Swedish horses [30]. The linear scaling of conformation provided better recognition of horse type conformation, as six traits out of twenty possible traits were different. Warmblood mares assessed as the jumping and dressage type showed differences of about 5–8% of the scale used. These differences should be studied in detail, as body conformation is recognized as one of the most important factors influencing lameness [31]. Only one movement trait was different in both types of mares—the balance of canter, being less balanced (7.16%) in jumping mares. The evaluation of only one movement trait may have prevented differentiation between types. It may be beneficial to evaluate a number of movement traits to provide data to differentiate between sport types. These differences were not evident in the overall points given by the judges for movement traits, as no trait from the overall point score was statistically significantly different between the sport types of mares. However, in specific kinds of gaits [32], even between racing and dressage horses, there may be a lack of differences.

Such low differences in horse conformation and movement of the sport types can be caused by small differentiation between jumping and dressage populations of horses. In Poland, the linear system and division of horses into different sport types started some years ago and might be biased by a lack of recognition by all breeders or owners. According to other researchers [27,28], it seems that type is not so restricted in the Dutch warmblood population (KWPN), as the existing unfavorable correlations are not strong. Earlier, Koenen et al. [2] stated that no larger differences exist between the conformation of good jumpers and good dressage horses. However, the specialization type of the horse accelerated negative correlations between traits, so the correlations may depend on this. Horses of different types differ in the amount of training, as jumping horses are prepared longer for young horses’ performance tests [4].

The other discussed item is the optimum for each sport horse type, as the position of the optimum is not necessarily in the middle or at the end of the scale—the optimum is subject to research [4,24]. The optimum for many breeds is not expressed in marks. Viklund and Eriksson [4] suggested that this may be connected with the risk that with optimum marking, the system will change into the traditional evaluation system. In some countries, the warmblood population is uniform, as are all sport horses, without specialization being identified [33]. According to the same author, some linear traits may overlap; thus, it is important to determine those traits that are close and those that are the same in the overall selection. It seems that the same is valid for different sport types of horses. It would be of the same importance concerning the population mean in linear scoring as that concerning the idea in traditional scoring. 

### 4.3. Breed and Stallion Effect

The stallion effect, expressed as repeatability, was mostly small for all the traits in all the systems. However, repeatability for the trait of “height at the withers” was on the medium level, and it was higher than that for the measured height at the withers from the biometric part of the evaluation. As reported earlier [21], measured traits and more objective traits [27,28] are less repeatable and heritable, because some of the variation may be due to judges’ knowledge of the catalogue and their subjectivity [9,27,28]. 

The same low level of repeatability was calculated for the other linear traits and the 100-point scaling traits, except for jumping traits, going up to 0.5. The preselection may affect repeatability as heritability [27,28]. The low level of genetic parameters may be caused by the effects, for which a disturbance cannot be adjusted. The effect of the sire-stallion may depend on the order of horse scoring. If horses by one sire are presented together as a group, the genetic component might be higher, as judges may take the sire value into account. This was not the case in our study, as horses were not presented by sire; however, the catalog with pedigree information was known. Except for the evaluation order, the fact that judges may potentially discuss, and thus influence, possibilities between each other is significant [4]. The type of presentation also influences trait evaluation [1]. 

The basic conformation measurements in individual breeds were different for all the investigated breeds, and the most differences were found between the Małopolska breed and the other breeds, which seems reasonable, as the Małopolski horse (Młp) is related to the Anglo-Arabian and Arabian horses. The breed effect was not significant for jumping traits, but this may be caused by limited jumping data. The breed effect was considered in the models as the thoroughbred percentage [27]. Even if the breed effect is used in some studies, the effect of the breed is not presented in detail [2,27,28].

### 4.4. Other Effects

In traditional scoring, a judge’s preferences may play a role [3], but in the linear system, the experience of a judge is a significant effect. Equal evaluation in every region is of great importance. In our study, the effect of the region is significant for all the investigated traits. However, it is very difficult to discuss this effect, as it is a multi-factorial effect. It can be connected with the quality of warmblood mares in a given district, but also with the quality of breeding in the individual regions. This quality of breeding can be related to the quality of the horse evaluation, the quality of the environment (pasture quality, but also duration of the pasture period), feeding practices and many other factors. Such effects are usually included in the statistical models as location/data/evaluating person/classifier [2,3,23,27,28,33,34]. The region (as the team of judges) is of special importance, as using appropriate scaling and reliability of results is crucial [23]. Similarly, in linear scoring, the classifier’s experience and the classifier’s mean of the population are extremely important; the flat shoulder in the Swedish warmblood will look different from the flat shoulder of the Pura Rasa Espanola [35]. Judges representing different disciplines may have different scaling methods [36], as jumping traits have larger residual variances [4]. Jumping is probably more influenced by the neighboring environment. Some researchers underline the fact that the traits evaluated in linear scaling can vary in accordance with the environmental conditions (in hand, loose, under the rider), whereas outstanding evaluators can see more differences in horse traits than owners, who used to their horses [24].

Comparable variance components for traditional and linear scoring (except for jumping traits) were found by Viklund and Eriksson [4]. Such results were also recorded in our study. The breed and district effects were the most significant for almost all the traits. The district was the most important for linear conformation traits, and the breed was most important for movement linear traits. The breed effect was not significant for the linear jumping traits, but this may be connected to the small amount of jumping data. The investigated type of the horse was significant for 33% of measured traits, 29% of linear conformation and 22% of the traditional points system. Movement traits differed for 10% of traits between the sport types of mares. The percentage of traits can be influenced by their number (e.g., only three body measurements). However, more objective measurements in the evaluation of horses are underlined as a possible next step [1,24]. More objective evaluation would benefit from automatic measurements of body conformation, movement and jumping; or these could at least be a basis for comparison [4]. Roth et al. [35] wrote that new measurements and their ratios can be used for horse type recognition. More measurements can be performed by new visual methods for living horses without stress for the horse.

### 4.5. Correlations

The correlations based on points (100-point scaling vs. general points in the linear description) could be expected to be high. Both point-scaling systems were correlated above meaningful 0.5 values only between the same named traits—type, movement and gait. Based on the recorded values, these traits cannot be treated as the same traits, because their connections are not strong enough. The expected value above 0.8 would allow us to treat the traits as having the same characteristics. Novotna et al. [33] also found low values of phenotypic correlations (often around zero) between the conformation and performance traits used for the entry of horses into the stud book. Medium–high phenotypic correlations were found by the cited authors only between quantitatively measured traits (height at the withers, heart girth, cannon bone circumference): 0.22–0.38, as well as between walk and trot stride lengths: 0.47. Genetic correlation was also estimated and showed significantly higher values, from −0.47 (nobleness and heart girth) to 0.92 (back length and length of loins). 

In this research, almost all phenotypic correlations between traits belonging to other evaluated systems were negative, which means that mares who scored closer to the left extremum (“a”) in linear profiling were assessed higher in the 100-point system. Similarly, in a study by Viklund and Eriksson [4], phenotypic and genotypic correlations between linear traits and corresponding traditionally scored traits were mainly negative, but most of them were high (in many cases, between −0.7 and −0.9). In turn, in other publications [27,28], phenotypic and genetic correlations of descriptive and subjective traits were first of all estimated at a low and medium level. 

The calculation of correlations seems to provide a direct comparison of these two systems, which are constructed in a rather different manner. One is based on a comparison of the horse, based on the idea of the correctly conformed horse and its valuation, and the other is designed as a description of the horse according to the population mean. Both systems depend on human experience and knowledge, as the former can differ in ideas, and the latter depends on the population. In any case, it can be expected that the direction of changes observed on the description diagram will follow valuation in points. Based on such an understanding, quite strong connections were noted for movement traits for walking and trotting (−0.7 and −0.6). This seems to come from the fact that movement traits in the linear description are also described from the best to the worst value. Such a connection in linear jumping traits was noted for the scope; however, it was on the lower level. Low correlations may be due to the non-linear nature of the trait connections. Very high and very low values may have an adverse effect on performance [2]. Some connections between traits may be indirect, as the uneven hoof is supposed to be connected with a long neck (mainly in foals); trait ratios can also influence other traits [37]. By evaluating horses and by discussing the meaning of correlations, it should be taken into account that traits may compensate for each other—the high height at the withers produces slower motion, but longer limbs compensate for this [38]. In turn, in sports with high accelerations and maneuverability, it is expected that a lower body size is preferable [38]. Even though this does not appear to be so in our study, such observations were noted between different types of Quarter horses [35]. A greater leg circumference in relation to body size gives better stability [38]. We found no differences between the circumference of the leg, but ratios between measurements should still be investigated.

Partial correlations evaluated for the same traits were less significant for investigated trait connections between systems. Corrections for the factors that were significant for the analysis of variance did not change the directions of connections, and the values of significant partial correlations were higher. However, even higher calculations did not reach the value of 0.8, being the index of the same trait.

### 4.6. Limitations 

Even though the amount of data was relatively large, only some of the investigated warmblood mares had data for all the movement traits, and a very small amount of the jumping information was available. Some of the lack of data is because the linear scoring system is new in Poland, and not all traits were evaluated simultaneously (before 2017, this system was used occasionally, and since 2019, most young mares have been evaluated). Thus, the statistical model could not be optimal. Further studies should solve this problem. For most traits examined, there were sufficient data to generate statistically relevant findings. Within the population examined, only the jumping traits were low in numbers, which may have restricted our ability to draw conclusions. Because most evaluated warmblood mares with both evaluations and measurements were young mares between 3 and 4 years old, the age effect was not investigated. The sex effect was not investigated because of the data selection caused by the data availability. However, information coming from that sample population can help to find differences between warmblood mares of different sport types. The influence of the classifiers was not incorporated directly into the statistical model. In the presented data, mares were judged by the judging committees that were included in the breeding district effect. 

Usually, linear profiling and point-based evaluation systems are studied from a genetic point of view, calculating heritabilities and genetic correlations [3,4,27,28,39,40]. However, breeders and geneticists should not forget that the genetic parameters and evaluations can only be as good as the data used for the calculations. The analysis showed that the horse sport type effect is a less recognizable factor in the linear evaluation. In any case, this still offers a better possibility to recognize the type compared to the traditional point system. This corresponds with the earlier research, as new, more objective methods are stated as useful for further research. According to our study, too, the standard deviations are lower than expected, and the range of extremes could be discussed and probably divided into more detailed scaling, as suggested by Viklund and Eriksson [4]. Because of low repeatability, the combined evaluation with points should help to cover all the various sources of information. Such a solution was suggested by the authors of [27,28], especially as scoring traits individually may help in providing an overall view of the horse in linear scoring, and extensive scoring reporting is highly appreciated by breeders [2]. 

The size and shape of the horse’s body are significant factors; the importance of every trait can be discussed, and traits have to be monitored continuously, as linear sheets are continuously changing and evolving. The most preferred method of presentation by breeders and evaluators is one in which all traits are analyzed jointly rather than traits being grouped; however, too much information could make the interpretation difficult and confusing [3]. Uniformity, as an indicator of the high quality of the analyzed data, is underlined in the linear evaluation, and a satisfactory level of selection is obtained without specific and expensive equipment [3]. Many traits can be managed by special applications created for breeders, which is very convenient [24]. Some applications can provide or consider specific indicators [33].

The linear scoring system offers a satisfactory judgment of heritability; thus, traditional scoring has been replaced by this scaling in many countries [3]. However, it seems that a problem may arise if not enough traits are considered, or their evaluation is not clear enough. The number of differences between types of mares is unexpectedly small. The small differences between the horse sport types can be explained by the judges’ corrections in the evaluation of the horse type. Obtained differences, however, are not always clear to understand. Unexpectedly, there are no differences in movement characteristics between horse groups for jumping and dressage. The question is whether the same descriptions of sport type populations mean the same to the evaluators. Further, more detailed analyses of measured traits are needed. 

The comparison of the 100-point evaluation and linear scoring systems with the use of simple correlations showed meaningful correlations only between the general traits in the linear assessment and 100-point valuation scores. It was observed in most cases that the higher the 100-point assessment, the further to the left the descriptive scale. Positive correlations were shown only in individual cases. None of the traits in the two evaluation systems is identical to any other (r > 0.8). Only the general traits in the linear and 100-point systems were related to each other at a level above the average. On the other hand, among the detailed descriptive assessments, only single traits showed a correlation power above the average. Other detailed measurements may be obtained to search for differences between sport horse types.

There is no doubt that summarizing traits into a single mark is not appropriate, because by averaging the traits, one will lose too much information, and the genetic evaluation will lose its reliability [33]. Some international comparisons can help in the proper recognition of traits [41], and international projects are being initiated [42]. Further, a more neutral/measurable assessment could be useful in judges’ training to ensure better identification of differences between individual sports groups or breeds. There are biomechanical data comparing the quality of jumping and dressage horses [43,44,45]; however, most of these describe adult, high-level horses. Investigations into trait definitions for young horses in basic training have also been performed [46,47]. However, some researchers deepen the analysis of linear traits, looking for clear differences in particular breeds or types of horses [48,49]. Others are looking for new measurements and methods of assessing the conformation of horses [50,51,52].

## 5. Conclusions

Because smaller differences than expected were found in both current evaluation systems—100 points and linear scoring—new traits describing the sport horse type in a more objective way are needed. This is especially important as the repeatability of traits in both systems is mostly low, so the genetic progress is limited. Only the jumping traits in the linear evaluation indicate the opportunity for adequate selection because of average repeatability values. The linear evaluation system gives more information on the sport type of warmblood sport mares than the traditional 100-point evaluation, which shows that it is a better option for use in breeding. Only general traits in the linear assessment and 100-point valuation scores are comparable using simple correlation. None of the traits in the two evaluation systems is identical to any other regardless of the calculation method; therefore, a transformation of the evaluation between systems will be more difficult. The differences between warmblood mares that are obtained by these systems are usable for the same kinds of evaluations.

## Figures and Tables

**Table 1:** Statistical characteristics of traits in 100-point evaluation system.

Trait	Scale (pts)	N	Mean	SD	Min.	Max.
Type	0–15	1547	13.40	0.56	12	15
Head and neck	0–5	1547	3.87	0.39	3	5
Body	0–10	1547	13.18	0.59	11	14
Forelegs	0–10	1546	6.41	0.53	5	8
Hind legs	0–10	1547	6.44	0.54	5	8
Hooves	0–10	1547	6.83	0.53	5	9
Walk	0–10	1547	7.26	0.58	5	9
Trot	0–10	1547	7.13	0.58	6	9
General impression	0–15	1547	13.28	0.51	12	14

SD—standard deviation; N—number.

**Table 2:** Statistical characteristics of linear general traits.

Trait (0–10 pts)	N	Mean	SD	Min.	Max.
Movement	657	6.90	0.67	5.0	9.0
Conformation	1196	6.83	0.75	5.0	9.5
Type	854	7.16	0.77	5.0	9.5
Walk	904	6.92	0.72	5.0	9.0
Trot	856	6.89	0.78	4.0	9.5
Canter	535	6.71	0.70	5.0	9.0
Free-jumping	121	7.09	1.02	4.5	9.5
Reflex	119	7.01	1.01	4.5	9.5
Technique	119	6.99	1.01	4.5	9.5
Abilities	119	7.03	1.08	4.5	9.5

SD—standard deviation, N—number.

**Table 3:** Statistical characteristics of linear conformation traits.

	Trait (1–40 pts)	a/1	i/40	N	Mean (pts)	SD	Min.	Max.
**Conformation**	Body shape	rectangular	square	1547	13.55	5.49	1.0	35.0
	Body direction	uphill	downhill	1547	23.27	5.79	5.0	40.0
	Head and neck connection	light	heavy	1547	18.70	6.89	5.0	40.0
	Length of neck	long	short	1547	18.68	6.66	5.0	35.0
	Position of neck	vertical	horizontal	1547	21.45	7.07	1.0	40.0
	Muscling of neck	heavy	poor	1547	20.81	7.31	5.0	40.0
	Height of withers	high	flat	1547	16.79	7.05	1.0	35.0
	Length of withers	long	short	1026	17.77	6.16	1.0	35.0
	Position of shoulder	sloping	straight	1547	21.17	7.14	5.0	40.0
	Line of back	roached	weak	1547	22.92	5.33	5.0	40.0
	Line of loins	roached	weak	1547	20.95	6.76	1.0	40.0
	Shape of croup	sloping	flat	1547	15.71	6.17	1.0	35.0
	Length of croup	long	short	1547	21.35	6.60	5.0	40.0
	Stance of forelegs	over at the knee	back at the knee	1547	21.88	5.07	5.0	40.0
	Stance of hind legs	sickle hocked	straight	1544	19.68	6.74	1.0	40.0
	Stance of pastern front	weak	upright	1547	19.01	5.98	1.0	35.0
	Stance of pastern hind	weak	upright	1026	18.15	5.27	5.0	35.0
	Shape of hooves	wide	narrow	1547	20.72	7.09	1.0	40.0
	Heels	high	low	1547	22.71	6.69	1.0	40.0
	Quality of legs	lean	blurred	1545	16.25	7.44	1.0	40.0
	Substance of legs	heavy	fine	1546	24.21	6.43	5.0	40.0
**Gaits**	Walk length of stride	long	short	1292	17.55	7.22	1.0	40.0
	Walk correctness	toed in	toed out	1296	19.27	5.66	1.0	35.0
	Trot length of stride	long	short	1133	19.12	7.07	5.0	40.0
	Trot elasticity	elastic	stiff	1130	20.15	7.42	1.0	40.0
	Trot impulsion	powerful	weak	1095	18.52	7.73	5.0	40.0
	Balance	carrying	pushing	1052	19.93	7.44	1.0	40.0
	Canter length of stride	long	short	636	22.13	6.83	5.0	40.0
	Canter elasticity	elastic	stiff	412	21.99	6.90	5.0	40.0
	Canter impulsion	powerful	weak	631	19.86	7.25	1.0	40.0
	Canter balance	carrying	pushing	631	22.64	7.02	5.0	40.0
**Jumping**	Take off: direction	upwards	forwards	139	22.12	7.44	5.0	40.0
	Take off: quickness	quick	slow	139	20.51	8.26	1.0	35.0
	Technique: forelegs	bent	streached	139	20.00	8.96	1.0	40.0
	Technique: hind legs	rounded	hollow	139	20.85	8.51	1.0	40.0
	Technique: haunches	open	fixed	139	20.37	8.09	1.0	35.0
	Scope	much	little	139	20.69	9.66	1.0	40.0
	Elasticity	supple	stiff	139	22.17	9.39	1.0	40.0
	Care	careful	not careful	139	18.02	8.19	1.0	40.0
	Attitude	much	little	138	19.40	8.44	1.0	35.0

SD—standard deviation, N—number.

**Table 4:** LS means and standard errors (LSM, SE) of biometrical traits for sport type classification.

Trait (N)	Sport Type Classification LSM (SE)	
	DH	JH
Height at withers (N = 1547)	164.46 (0.21)	164.23 (0.26)
Chest circumference (N = 1547)	194.49 ^A^ (0.40)	192.68 ^A^ (0.50)
Cannon circumference (N = 1547)	20.50 (0.04)	20.40 (0.05)

A—the same letters indicate statistically significant differences, with capital letters for *p* ≤ 0.01; DH—dressage horse, JH—jumping horse.

**Table 5:** LS means and standard errors (LSM, SE) for sport type classification.

Trait (N)	Sport Type Classification LSM (SE)	
	DH	JH
Type (N = 1547)	13.44 (0.04)	13.54 (0.04)
Head and neck (N = 1547)	3.89 (0.03)	3.88 (0.03)
Body (N = 1547)	13.24 (0.03)	13.19 (0.03)
Forelegs (N = 1546)	6.39 ^a^ (0.03)	6.30 ^a^ (0.03)
Hind legs (N = 1547)	6.40 (0.03)	6.34 (0.03)
Hooves (N = 1547)	6.85 (0.03)	6.80 (0.04)
Walk (N = 1547)	7.31 ^a^ (0.03)	7.22 ^a^ (0.04)
Trot (N = 1547)	7.12 (0.03)	7.14 (0.04)
General impression (N = 1547)	13.35 (0.03)	13.32 (0.03)

a—the same letters indicate statistically significant differences, small letters for *p* ≤ 0.05; DH—dressage horse, JH—jumping horse.

**Table 6:** LS means and standard errors (LSM, SE) for sport type.

Trait (N)	Sport Type Classiffication LSM (SE)	
	DH	JH
Movement (N = 657)	6.78 (0.07)	6.78 (0.08)
Conformation (N = 1196)	6.73 (0.06)	6.81 (0.07)
Type (N = 854)	6.95 (0.07)	7.00 (0.07)
Walk (N = 904)	6.85 (0.07)	6.77 (0.08)
Trot (N = 856)	6.74 (0.07)	6.69 (0.08)
Canter (N = 535)	6.71 (0.09)	6.70 (0.10)

DH—dressage horse, JH –jumping horse.

**Table 7:** LS means and standard errors (LSM, SE) of jumping linear traits depending on the sport type classification.

Trait (N)	Sport Type LSM (SE)	
	DH	JH
Position of neck (N = 1547)	21.95 ^a^ (0.36)	20.89 ^a^ (0.45)
Position of shoulder (N = 1547)	21.81 ^A^ (0.30)	20.33 ^A^ (0.44)
Line of loin (N = 1547)	21.04 ^A^ (0.34)	22.24 ^A^ (0.43)
Shape of croup (N = 1547)	16.96 ^A^ (0.31)	15.74 ^A^ (0.39)
Stance of pastern (N = 1547)	18.20 ^a^ (0.28)	19.17 ^a^ (0.38)
Quality of legs (N = 1545)	15.51 ^a^ (0.37)	16.55 ^a^ (0.46)

A, a—the same letters indicate statistically significant differences, with capital letters for *p* ≤ 0.01 and small letters for *p* ≤ 0.05; DH—dressage horse, JH—jumping horse.

**Table 8:** LS means and standard errors (LSM, SE) of movement linear traits depending on the sport type of horse.

Gait (N)	Trait	Sport Type LSM (SE)	
		DH	JH
Canter (N = 631)	Balance	21.40 ^a^ (0.07)	23.03 ^a^ (0.07)

a—the same letters indicate statistically significant differences, small letters for *p* ≤ 0.05; DH—dressage horse, JH—jumping horse.

**Table 9:** Correlations between general linear scoring traits and 100-point assessment.

Trait	Type	Head and Neck	Body	Forelegs	Hind Legs	Hooves	Walk	Trot	General Impression
Movement	0.28	0.08	0.09	0.16	ns	0.50	0.57	0.57	0.25
Conformation	0.43	0.25	0.35	0.31	0.32	0.13	0.22	0.33	0.36
Type	0.52	0.18	0.19	0.18	0.22	0.08	0.18	0.32	0.30
Walk	0.18	ns	0.13	0.07	0.11	ns	0.67	0.29	0.16
Trot	0.22	0.07	0.16	0.11	0.14	ns	0.21	0.66	0.22
Canter	0.23	0.09	0.19	ns	0.17	ns	0.23	0.43	0.19
Free-jumping	0.33	0.21	0.28	ns	0.18	ns	ns	0.29	0.35
Reflex	0.28	0.18	0.26	ns	0.20	ns	ns	0.25	0.34
Technique	0.32	0.29	0.23	ns	ns	ns	ns	0.17	0.33
Abilities	0.33	0.16	0.22	ns	ns	ns	ns	0.26	0.30

Note: ns—non-significant; the other traits are significant for *p* ≤ 0.05.

**Table 10:** Correlations between linear conformation grades and 100-point evaluation.

Trait	Type	Head and Neck	Body	Forelegs	Hind Legs	Hooves	Walk	Trot	General Impression
Body shape	0.06	ns	ns	ns	ns	ns	ns	ns	ns
Body direction	−0.21	−0.13	−0.15	−0.06	−0.07	ns	ns	ns	−0.19
Head and neck connection	−0.14	−0.24	ns	ns	ns	0.09	ns	ns	ns
Length of neck	−0.18	−0.32	−0.08	ns	ns	ns	−0.08	−0.07	−0.13
Position of neck	−0.15	−0.16	−0.09	ns	ns	ns	−0.05	−0.18	−0.13
Muscling of neck	−0.16	−0.21	−0.22	−0.09	ns	ns	−0.06	−0.17	−0.24
Height of withers	−0.07	ns	0.07	ns	ns	ns	ns	ns	ns
Length of withers	−0.11	ns	ns	ns	ns	ns	ns	ns	ns
Position of shoulder	−0.24	−0.15	−0.17	−0.06	ns	−0.05	−0.13	−0.16	−0.18
Line of back	−0.08	ns	−0.25	ns	−0.05	−0.05	ns	ns	−0.06
Line of loins	−0.15	−0.09	−0.27	ns	ns	ns	−0.09	−0.07	−0.15
Shape of croup	ns	ns	0.09	ns	ns	ns	ns	ns	ns
Length of croup	−0.19	−0.11	−0.26	ns	ns	ns	−0.08	−0.08	−0.17
Stance of forelegs	−0.05	−0.05	ns	−0.15	ns	0.06	ns	ns	ns
Stance of hind legs	ns	ns	ns	ns	0.15	ns	ns	−0.05	ns
Stance of pastern hindleg	ns	ns	ns	ns	0.21	0.06	ns	ns	0.08
Shape of hooves/feet	−0.07	ns	ns	−0.10	0.06	−0.45	−0.11	−0.10	−0.06
Heels	−0.05	ns	ns	ns	−0.02	−0.14	ns	ns	ns
Quality of legs	−0.07	−0.05	ns	ns	−0.09	−0.07	−0.11	−0.08	−0.09
Substance of legs	ns	ns	−0.07	−0.11	ns	−0.13	ns	ns	ns

Note: ns—non-significant; the other traits are significant for *p* ≤ 0.05.

**Table 11:** Correlations between linear movement traits and 100-point assessment.

Trait	Type	Head and Neck	Body	Forelegs	Hind Legs	Hooves	Walk	Trot	General Impression
Walk: Length of stride	−0.13	−0.06	−0.05	ns	ns	ns	−0.66	−0.21	−0.07
Walk: Correctness	0.09	−0.06	ns	ns	ns	ns	ns	−0.06	ns
Trot: Length of stride	−0.28	−0.11	−0.19	ns	ns	ns	−0.22	−0.60	−0.17
Trot: Elasticity	−0.27	−0.15	−0.16	−0.07	ns	ns	−0.23	−0.53	−0.19
Trot: Impulsion	−0.24	−0.18	−0.19	−0.06	−0.09	ns	−0.16	−0.52	−0.24
Trot: Balance	−0.28	−0.14	−0.15	ns	ns	ns	−0.21	−0.47	−0.24
Canter: Length of stride	−0.28	−0.15	−0.11	ns	−0.09	ns	−0.26	−0.29	−0.16
Canter: Elasticity	−0.21	−0.15	ns	ns	−0.14	ns	−0.27	−0.26	ns
Canter: Impulsion	−0.21	−0.18	−0.15	ns	−0.14	−0.08	−0.17	−0.33	−0.21
Canter: Balance	−0.28	−0.16	−0.17	ns	−0.08	−0.10	−0.23	0.33	−0.17

Note: ns—non-significant; the other traits are significant for *p* ≤ 0.05.

**Table 12:** Correlations between linear jumping scores and 100-point assessment traits.

Trait	Type	Head and Neck	Body	Forlegs	Hind Legs	Hooves	Walk	Trot	General Impression
Take off: Direction	−0.24	ns	−0.26	ns	ns	ns	ns	−0.20	−0.20
Take off: Quickness	−0.15	−0.22	−0.18	ns	ns	ns	ns	ns	−0.23
Technique: Forelegs	−0.26	−0.20	ns	ns	ns	ns	ns	ns	ns
Technique: Hind legs	−0.29	−0.23	−0.26	ns	ns	ns	ns	ns	−0.17
Technique: Haunches	−0.22	ns	−0.23	ns	ns	ns	ns	−0.20	−0.19
Scope	−0.30	ns	−0.24	ns	−0.19	ns	ns	−0.30	−0.28
Elasticity	0.26	−0.17	−0.24	ns	ns	ns	−0.25	ns	−0.23
Care	−0.24	ns	ns	ns	ns	ns	ns	ns	−0.18
Engagement	−0.20	ns	−0.24	ns	−0.18	ns	ns	ns	−0.17

Note: ns—non-significant, and the other traits are significant for *p* ≤ 0.05.

## Data Availability

Restrictions apply to the availability of these data. Data were obtained from PHBA and are available with the permission of PHBA.

## Supplementary Materials

The following supporting information can be downloaded at: https://www.mdpi.com/article/10.3390/ani13193101/s1, Table S1: Description of basic measurements; Table S2: Description of conformation traits in 100-point evaluation; Table S3: Description of overall evaluation in linear scoring; Table S4: Description of conformation traits in linear scoring; Table S5: Description of movement traits in linear scoring; Table S6:Description of jumping traits in linear scoring; Table S7: LS means and standard errors (LSM, SE) of biometrical traits for breeds; Table S8: LS means and standard errors (LSM, SE) of 100-points scoring traits for breeds; Table S9: LS means and standard errors (LSM, SE) of general linear traits for breeds; Table S10: LS means and standard errors (LSM, SE) of linear conformation traits in the horse breed; Table S11: LS means and standard errors (LSM, SE) of movement linear traits depending on the horse breed; Table S12: LS means and standard errors (LSM, SE) of movement linear traits depending on the breed of horse.
