# Supplementary material for: Comparison of Conformation and Movement Characteristics in Dressage and Jumping Sport Warmblood Mares Based on Point Evaluation and Linear Scoring System
Source: Animals (Basel). 2023 Oct 4;13(19):3101. doi: 10.3390/ani13193101 (PMC10571798; doi:10.3390/ani13193101)
Supplement: Supplementary file 1 [file animals-13-03101-s001.zip › animals-2580571-supplementary.pdf]

Supplementary Materials:

Table S1. Description of basic measurements (Zwoliński, 1980 [26]).

Trait (cm)	Description
Height at the withers	Measured at the highest point of the withers from the ground
Circumference of the cannon bone	Measured with a tape at the thinnest point
Circumference of the chest	Measured with a tape behind the rear edges of the shoulder blades

Table S2. Description of conformation traits in 100-point evaluation (Zwoliński, 1980 [26]).

Trait (pts)	Description
Type (0–15)	Distinct features of a breed and gender type
Head and neck (0–5)	Head and neck profile—breed characteristic, length and musculature
Body (0–10)	Shape typical of the breed and utility of horses (rectangular–square), depth and width of the chest (high-leggedness), correctness of the back profile
Front legs (0–10)	Correct posture and position in front and side, length and angulation of each part, musculature, joint dryness and tendon health, lesions, osteomas
Hind legs (0–10)	Correct posture and position in back and side, length and angulation of each part, musculature, joint dryness and tendon health, lesions, osteomas
Hooves (0–10)	Correct posture and shape in front, back and side, width, angulation of hooves and length of each wall, health, lesions
Walk (0–10)	Correctness, relaxation, regularity, length of stride, impulse
Trot (0–10)	Correctness, relaxation, regularity, length of stride, impulse
General impression (0–10)	Beauty, general harmony of structure, musculature, constitution appropriate to the type of horse

Table S3. Description of overall evaluation in linear scoring.

**Trait (0–10)**	**Description**
Conformation	Correct posture, profile and shape of each part, assessment of conformation defects
Type	Correct for breed and gender type
Movement	Overall evaluation for walk, trot and canter
Walk	Correctness, relaxation, regularity, length of stride, impulse
Trot	Correctness, relaxation, regularity, length of stride, impulse
Canter	Correctness, relaxation, regularity, length of stride, impulse
Free-jumping	Technique, abilities and reflex of jump
Reflex	Jump quickness and atitiude
Technique	Correctnes of jump (take off and landing), position of forelegs, hind legs and haunches
Abillities	Scope of jump, jump without mistakes, altitude

https://kwpn-na.org/keurings/scoring-procedures/linear-scoring/ (accessed on 30 September 2023).

Table S4. Description of conformation traits in linear scoring.

**Conformation Trait**	**Description**
Body shape (rectangular–square)	A horse is rectangular when the length (from the point of the shoulder to the point of the buttock) is greater than the height. Horses need rectangular conformation in order to be athletic and elastic. A horse is square when the length of the horse is equal to the height of the withers.
Body direction (uphill–downhill)	The direction of the body as compared to the horizontal. A horse is harder to collect when built on the forehand (downhill).
Head and neck connection (light–heavy)	This trait is actually divided in two: the length of the poll and the throatlatch. Light: a long poll, light throatlatch and small distance between the top and bottom of the neck. Heavy: a short poll, a heavy throatlatch and a larger distance between the top and bottom of the neck. A lighter connection is desirable for acceptance of the bit.
Length of neck (long–short)	The distance from the poll to the peak of the withers (relative to the rest of the topline).
Position of neck (vertical–horizontal)	The angle that the neck makes with the horizontal when the horse stands naturally. Dressage horses need a more vertical position of the neck for easy self-carriage and uphill balance. Jumpers need a more horizontal position of the neck in order to collect themselves just before and during take off.
Muscling of neck (heavy–poor)	The measure of muscling in the neck, especially in the topline. Heavy: slight cresting of a thick neck. A rounded topline is a matter of muscling. Poor: thin neck, often with weak connection of topline into the withers.
Height of withers (high–flat)	The height of the withers measured as the distance between two horizontal lines—one through the peak of the withers, the other through the base of the withers. The height of the withers is important for a good saddle placement.
Length of withers	The length of the withers is measured by a horizontal line between the highest and lowest point of the withers.
Position of shoulder (sloping–straight)	The angle of the shoulder with the horizontal.

Line of back (roached–weak)	The course of the topline between the withers and the loins. A smooth, well-muscled topline enables the horse to move with good use of the back.
Line of loins (roached–weak)	The course of the topline from the back to the croup. Strong muscling is needed to stretch the back and hindquarters during the second phase of the jump.
Shape of croup (sloping–flat)	The angle from the point of the hip to the point of the buttock. Too much slope hinders flexibility of the pelvis, which is needed for collection, especially in piaffe and passage.
Length of croup (long–short)	The length from the point of the hip to the point of the buttock. The length is important for proportional conformation: the forehand, middle and hindquarters should form equal sections.
Stance of forelegs (over at the knee–back at the knee)	The angle made by lines drawn through the forearm and cannon, as seen from the side. The stance of the forelegs should be straight to ensure durability.
Stance of hind legs (sickle hocked–straight)	The angle made by lines drawn through the gaskin and cannon, as seen from the side. The hock angle should be approximately 150 degrees.
Stance of pastern front (weak–upright)	The angle made by the pasterns of the forelegs with the ground. A pastern that is too short and/or upright is more susceptible to concussive injury; a pastern that is long and too sloped (”soft” or ”weak”) is more easily strained.
Stance of pastern hind # (weak–upright)	The angle made by the pasterns of the hindlegs with the ground. A pastern that is too short and/or upright is more susceptible to concussive injury; a pastern that is long and too sloped (“soft” or “weak”) is more easily strained.
Shape of hooves (wide–narrow)	The relation between the coronet band and the carrying surface viewed from the front. Note: with unequal hooves, the narrow hoof is considered to be abnormal. The horse’s feet should be large and symmetrical.
Heels (high–low)	The height of the heels of the front hooves, measured between the ground and the coronet band. The heels are responsible for the correct position of the foot and pastern. Heels that are broad and moderately high are best for soundness.
Quality of legs (lean–blurred)	The extent to which the hindlegs are free from blemishes or swelling. Lean legs show soundness and general quality.
Substance of legs (heavy–fine)	The circumference of the leg, including the size of the joints, relative to the size of the horse’s body. A horse needs well-developed bones, joints and tendons in order to be durable.

https://kwpn-na.org/keurings/scoring-procedures/linear-scoring/(accessed on 30 September 2023).

Table S5. Description of movement traits in linear scoring.

Gait	Movement Trait	Description
Walk	Length of stride (long–short)	The length of the stride.
	Correctness (toed-in–toed-out)	The walk evaluated from the front. When the hooves turn in from the fetlock, they are considered to be toed-in. When the hooves turn out from the fetlock, they are toed-out.

Trot	Length of stride (long–short)	The length of the stride.
	Elasticity (elastic–stiff)	The measure of elasticity with which the movement flows through the body and is then absorbed by the legs.
	Impulsion (powerful–weak)	The measure in which the horse pushes off the ground with the hindlegs.
	Balance (carrying–pushing)	The combination of body position and the measure in which the hindleg carries.
Canter	Length of stride (long–short)	The length of the stride.
	Elasticity (elastic–stiff)	The measure of elasticity with which the movement flows through the body and is then absorbed by the legs.
	Impulsion (powerful–weak)	The measure in which the horse pushes off the ground with the hindlegs.
	Balance (carrying–pushing)	The combination of body position and the measure in which the hindleg carries.

https://kwpn-na.org/keurings/scoring-procedures/linear-scoring/(accessed on 30 September 2023).

Table S6. Description of jumping traits in linear scoring.

**Jumping Trait**	**Description**
Take off: direction (upwards–forwards)	The measure in which the horse is able to elevate the withers.
Take off: quickness (quick–slow)	In the last canter stride before the jump—the time between when the forelegs and the hindlegs hit the ground and the moment when the horse leaves the ground.
Technique: forelegs (bent–stretched)	The measure in which the shoulder bends, in combination with the upper arm, forearm and the cannon; “tuck.”
Technique: hind legs (rounded–hollow)	The measure in which the neck and back follow the arc over the jump; “bascule.”
Technique: haunches (open–fixed)	The measure in which the angles of the haunches are able to open during the second phase of the jump.
Scope (much–little)	The ability of the horse to jump upward with power, while at the same time developing a forward direction.
Elasticity (supple–stiff)	The measure of fluidity in which the entire jump (including the landing) flows because of elasticity in the body.
Care (careful–not careful)	The natural ability of the horse to jump without mistakes; the horse’s desire not to knock down a rail.
Attitude # (much–little)	The attitude of the horse to jump with power.

https://kwpn-na.org/keurings/scoring-procedures/linear-scoring/ (accessed on 30 September 2023).

trait added in Polish regulation.

Table S7. LS means and standard errors (LSM, SE) of biometrical traits for breeds.

Trait LSM (SE)	Breed			
	**m**	**Wlkp**	**sp**	**Other**
Height at withers	162.08 ABC (0.31)	164.86 A (0.30)	165.06 B (0.24)	165.37 C (0.34)
Chest circumference	190.16 ABC (0.60)	194.60 A (0.60)	194.91 B (0.44)	194.65 C (0.65)
Cannon circumference	19.89 ABC (0.31)	20.56 A (0.06)	20.70 B (0.05)	20.62 C (0.07)

A, a—the same letters indicate statistically significant differences, with capital letters for $p \leq 0.01$ and small letters for $p \leq 0.05$. Breeds: m—Małopolska horse, wlkp—Wielkopolska horse, sp—Polish sport horse, other—foreign breeds.

Table S8. LS means and standard errors (LSM, SE) of 100-points scoring traits for breeds.

Trait LSM (SE)	Breed			
	**m**	**Wlkp**	**sp**	**Other**
Type	13.46 ab (0.04)	13.32 aC (0.04)	13.39 D (0.03)	13.59 bCD (0.04)
Head and neck	3.87 (0.03)	3.85 (0.03)	3.86 (0.02)	3.94 (0.03)
Body	13.23 (0.04)	13.16 (0.04)	13.23 (0.03)	13.23 (0.05)
Forelegs	6.35 (0.04)	6.32 (0.04)	6.31 (0.03)	6.40 (0.05)
Hind legs	6.43 ab (0.04)	6.31 ac (0.04)	6.33 bd (0.03)	6.42 cd (0.04)
Hooves	6.81 (0.04)	6.81 (0.04)	6.82 (0.03)	6.87 (0.05)
Walk	7.16 AB (0.04)	7.26 (0.04)	7.30 A (0.03)	7.33 B (0.04)
Trot	7.09 A (0.04)	7.04 bC (0.04)	7.12 bD (0.03)	7.26 ACD (0.04)
General impression	13.34 a (0.04)	13.24 abc (0.04)	13.34 bd (0.03)	13.41 Cd (0.04)

A, a—the same letters indicate statistically significant differences, with capital letters for $p \leq 0.01$ and small letters for $p \leq 0.05$. Breeds: m—Małopolska horse, wlkp—Wielkopolska horse, sp—Polish sport horse, other—foreign breeds.

Table S9. LS means and standard errors (LSM, SE) of general linear traits for breeds.

Trait LSM (SE)	Breed			
	**m**	**wlkp**	**sp**	**other**
Movement	6.59 ^{abC} (0.09)	6.74 ^{aD} (0.09)	6.80 ^{bE} (0.08)	6.59 ^{CDE} (0.09)
Conformation	6.65 ^{A} (0.08)	6.67 ^{B} (0.07)	6.73 ^{C} (0.06)	7.01 ^{ABC} (0.08)
Type	6.96 (0.09)	6.90 ^{A} (0.08)	6.90 ^{B} (0.07)	7.16 ^{AB} (0.09)
Walk	6.78 (0.09)	6.76 (0.08)	6.81 (0.07)	6.90 (0.09)
Trot	6.60 ^{A} (0.09)	6.63 ^{B} (0.08)	6.67 ^{C} (0.08)	6.97 ^{ABC} (0.10)
Canter	6.63 ^{a} (0.13)	6.56 ^{B} (0.11)	6.66 ^{C} (0.10)	6.96 ^{aBC} (0.10)
Free-jumping	7.81 (0.80)	7.19 (0.31)	7.32 (0.28)	7.70 (0.38)
Reflex	7.68 (0.75)	7.03 (0.24)	7.14 (0.21)	7.56 (0.30)
Technique	7.94 (0.75)	7.07 (0.24)	7.14 (0.21)	7.39 (0.31)
Abilities	7.37 (0.80)	7.04 (0.26)	7.12 (0.30)	7.64 (0.32)

A, a—the same letters indicate statistically significant differences, with capital letters for $p \leq 0.01$ and small letters for $p \leq 0.05$. Breeds: m—Małopolska horse, wlkp—Wielkopolska horse, sp—Polish sport horse, other—foreign breeds.

Table S10. LS means and standard errors (LSM, SE) of linear conformation traits in the horse breed.

Trait LSM (SE)	Breed			
	**m**	**Wlkp**	**sp**	**Foreign**
Body shape	14.36 ^{aB} (0.40)	13.37 ^{c} (0.40)	13.38 ^{ad} (0.31)	12.23 ^{Bcd} (0.40)
Body direction	23.14 ^{A} (0.40)	23.11 ^{B} (0.41)	22.78 ^{C} (0.31)	21.00 ^{ABC} (0.46)
Head and neck connection	18.76 ^{a} (0.44)	18.83 ^{B} (0.50)	18.69 ^{C} (0.39)	17.04 ^{aBC} (0.60)
Length of neck	19.05 ^{a} (0.50)	18.47 ^{B} (0.49)	18.82 ^{C} (0.40)	17.27 ^{abC} (0.55)
Position of neck	22.42 ^{A} (0.53)	22.04 ^{B} (0.51)	21.65 ^{C} (0.40)	19.61 ^{ABC} (0.58)
Muscling of neck	22.58 ^{aB} (0.55)	22.12 ^{cd} (0.54)	21.02 ^{ac} (0.41)	20.33 ^{Bd} (0.40)
Height of withers	17.71 (0.52)	17.31 (0.51)	17.44 (0.40)	16.40 (0.58)
Length of withers	18.42 (0.52)	18.48 (0.53)	18.25 (0.39)	18.24 (0.56)
Position of shoulder	20.49 (0.52)	21.88 (0.51)	21.44 (0.40)	20.46 (0.40)

Line of back	22.77 (0.39)	23.48 (0.39)	22.97 (0.30)	22.93 (0.40)
Line of loins	22.39 [a] (0.51)	21.67 (0.49)	21.21 [a] (0.40)	21.30 (0.56)
Shape of croup	16.05 (0.46)	16.06 (0.44)	16.71 (0.34)	16.57 (0.50)
Length of croup	21.87 (0.49)	20.59 (0.48)	20.68 (0.37)	20.07 (0.55)
Stance of forelegs	21.71 (0.38)	22.03 (0.37)	22.17 (0.29)	22.14 (0.42)
Stance of hindlegs	17.79 [ab] (0.45)	18.73 (0.44)	19.30 [a] (0.34)	18.92 [b] (0.49)
Stance of pastern front	18.32 (0.45)	17.72 (0.46)	18.12 (0.33)	17.21 (0.48)
Stance of pastern hind	22.42 (0.54)	20.58 (0.53)	20.99 (0.41)	20.35 (0.60)
Shape of hooves	23.94 (0.50)	24.08 (0.48)	23.98 (0.37)	23.45 (0.54)
Heels	14.28 [aBC] (0.54)	15.69 [ade] (0.53)	16.99 [Bd] (0.40)	17.16 [ce] (0.60)
Quality of legs	14.27 [aBC] (0.54)	15.69 [aD] (0.52)	16.99 [BDe] (0.40)	17.15 [Ce] (0.59)
Substance of legs	26.12 [ABC] (0.49)	24.29 [A] (0.46)	23.75 [B] (0.36)	23.92 [C] (0.53)

A, a—the same letters indicate statistically significant differences, with capital letters for $p \le 0.01$ and small letters for $p \le 0.05$. Breeds: m—Małopolska horse, wlkp—Wielkopolska horse, sp—Polish sport horse, other—foreign breeds.

Table S11. LS means and standard errors (LSM, SE) of movement linear traits depending on the horse breed.

**Gait LSM (SE)**	**Trait**	**Breed**			
		**m**	**Wlkp**	**sp**	**Foreign**
trot	Elasticity	21.04 [a] (0.68)	21.51 [B] (0.65)	20.73 (0.52)	18.89 [aB] (0.76)
	Balance	20.66 (0.70)	21.83 [A] (0.69)	21.46 [B] (0.56)	19.46 [AB] (0.78)
Canter	Elasticity	22.90 [a] (1.29)	21.37 [b] (1.25)	21.07 (1.13)	18.65 [ab] (1.39)
	Balance	22.91 [a] (1.13)	22.94 [b] (1.02)	22.71 (0.94)	20.30 [ab] (1.19)

A, a—the same letters indicate statistically significant differences, with capital letters for $p \le 0.01$ and small letters for $p \le 0.05$. Breeds: m—Małopolska horse, wlkp—Wielkopolska horse, sp—Polish sport horse, other—foreign breeds.

Table S12. LS means and standard errors (LSM, SE) of movement linear traits depending on the breed of horse.

Trait	Breed			
	**m**	**wlkp**	**sp**	**Foreign**
Take off: direction	20.14 (3.40)	21.01 (1.77)	21.63 (1.56)	22.46 (2.18)
Take off: quickness	18.58 (3.79)	21.67 (1.97)	21.45 (1.74)	18.58 (3.79)
Technique: forelegs	16.82 (3.96)	19.72 (2.10)	19.50 (1.84)	19.03 (2.56)
Technique: hind legs	14.51 (3.86)	21.67 (1.98)	18.79 (1.75)	21.78 (2.47)
Technique: haunches	18.96 (3.67)	21.91 (1.90)	18.54 (2.35)	19.50 (2.35)
Scope	17.83 (4.44)	20.79 (2.30)	20.89 (2.03)	18.54 (2.85)
Elasticity	19.80 (4.39)	22.49 (2.25)	21.39 (1.99)	21.31 (2.82)
Care	18.31 (3.54)	18.66 (1.86)	17.33 (1.64)	15.37 (2.28)
Attitude	17.14 (4.09)	19.44 (2.07)	18.76 (1.84)	16.08 (2.79)

A, a—the same letters indicate statistically significant differences:,with capital letters for $p \leq 0.01$ and small letters for $p \leq 0.05$. Breeds: m—Małopolska horse, wlkp—Wielkopolska horse, sp—Polish sport horse, other—foreign breeds..
